# Autochthonous *Angiostrongylus cantonensis* Lungworms in Urban Rats, Valencia, Spain, 2021

**DOI:** 10.3201/eid2812.220418

**Published:** 2022-12

**Authors:** María Teresa Galán-Puchades, Mercedes Gómez-Samblás, Antonio Osuna, Sandra Sáez-Durán, Rubén Bueno-Marí, Màrius V. Fuentes

**Affiliations:** University of Valencia, Burjassot-Valencia, Spain (M.T. Galán-Puchades, S. Sáez-Durán, R. Bueno-Marí, M.V. Fuentes);; University of Granada, Granada, Spain (M. Gómez-Samblás, A. Osuna);; Laboratorios Lokímica, Catarroja-Valencia, Spain (R. Bueno-Marí)

**Keywords:** Angiostrongylus cantonensis, lungworm, Rattus norvegicus, Rattus rattus, rats, Valencia, Spain, parasites

## Abstract

To determine the role of rats as potential reservoirs of zoonotic parasites, we examined rats trapped in urban sewers of Valencia, Spain, in 2021. Morphologic and molecular identification and sequencing identified autochthonous *Angiostrongylus cantonensis* nematodes, the most common cause of human eosinophilic meningitis, in pulmonary arteries of *Rattus norvegicus* and *R. rattus* rats.

In Valencia, Spain, permanent rodent control campaigns are the responsibility of The Pest Control Section of the Health Service of Valencia City Council. As part of its tasks, the Section traps *Rattus norvegicus* and *R. rattus* rats in standard snap traps in the sewage system of Valencia. The trapped rodents were preserved in their entirety at −20°C and subsequently, to determine the potential reservoir role of zoonotic parasitic diseases, we defrosted the rats and analyzed the endoparasites.

In 2021, we collected 29 adult *A. cantonensis* nematodes (21 female and 8 male) from the organs of the first 27 trapped rats (25 *R. norvegicus* and 2 *R. rattus*) under a stereomicroscope once the rats had been dissected. The nematodes were detected in the pulmonary arteries of 2 *R. norvegicus* rats and 1 *R. rattus* rat; 7 young nematode adults were also found in the brain of the same *R. rattus* rat. Adult females showed the typical barber pole spiral of lungworms of the genus *Angiostrongylus* (Figure, panel A). After clarifying adult male worms with Amman’s lactophenol and studying their morphology (Figure, panels B–D), we found that the measurements were consistent with rat lungworm species of *A. cantonensis* (Table) (*1*,*2*).

**Figure:**
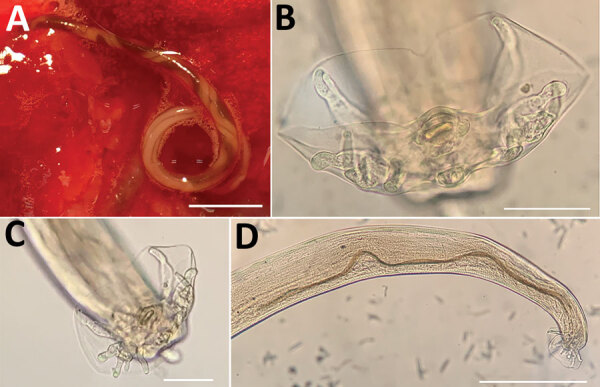
*Angiostrongylus cantonensis* lungworms from lungs of rats trapped in Valencia, Spain, 2021. A) Adult female with characteristic barber-pole appearance. Scale bar indicates 500 μm. B, C) Copulatory bursae of 2 male worms supported by bursal rays. Scale bars indicate 50 μm. D) Detail of the long spicula of a male worm. Scale bar indicates 300 μm.

**Table:** Measurements of 4 *Angiostrongylus cantonensis* male lungworms collected from rats trapped in Valencia, Spain, 2021*

Morphologic character	Range, mm	Mean, mm
Total length	14.08–21.08	17.21
Maximum width	0.25–0.38	0.28
Esophagus length	0.29–0.36	0.32
Esophagus maximum width	0.07–0.09	0.08
Distance from excretory pore to cephalic extremity	0.38–0.47	0.41
Spicules length	1.14–1.27	1.21
Gubernaculum length	0.10–0.13	0.11

*one from *Rattus norvegicus* and 3 from *R. rattu*s rats.

The parasite morphology, its microhabitat, and the nature of the definitive hosts clearly suggested that the parasites were *A. cantonensis.* To confirm species identification, we isolated total genomic DNA by using the DNeasy Blood and Tissue kit (QIAGEN, https://www.qiagen.com) according to the manufacturer’s instructions. We confirmed nematode species identity by PCR and sequencing of the cytochrome c oxidase subunit 1 (*3*); all sequences obtained were clustered with *A. cantonensis*. The phylogenetic tree grouped the *A. cantonensis* lungworms from Valencia close to the published sequences MK570629 and MN227185, corresponding to *A. cantonensis* lungworms isolated from Tenerife and Mallorca, respectively (Appendix Figure 1). We submitted the sequences we obtained to GenBank (accession no. ON819883 for the female specimen and ON819884 for the male). Likewise, when we sequenced the second internal transcribed spacer region, we found that our specimens formed a clade that differed from the other species of *Angiostrongylus* (Appendix Figure 2). We also submitted those sequences to GenBank (accession no. OM829831 for the male specimen and OM829832 for the female).

Male and female adult *A. cantonensis* lungworms live in the pulmonary arteries of *Rattus* rats, their preferred definitive hosts (*4*). Intermediate hosts are terrestrial or freshwater mollusks, such as snails and slugs. The female worms lay eggs, which give rise to L1 larvae that penetrate the alveolae and are swallowed by the rat and shed in the feces. After ingestion by an intermediate host, L1 larvae molt into L3 larvae. When infected mollusks are ingested by a rat, the subsequent phase takes place in the rat brain, where L3 larvae turn into young adults (L5). After leaving the central nervous system, L5 young adult worms reach the pulmonary arteries, where they mature and reproduce. Paratenic hosts include crabs, shrimp, frogs, and lizards (*4*).

Angiostrongyliasis is a foodborne disease; therefore, human infection requires ingestion of raw/poorly cooked intermediate or paratenic hosts. Another source of infection is lettuce contaminated with infective larvae released by an intermediate host (*5*). Hence, when humans accidentally ingest L3 larvae, the larvae penetrate the intestinal wall and travel through the bloodstream to the brain, where they can cause acute eosinophilic meningitis (neuroangiostrongyliasis). Severe cases can result in radiculitis, cranial neuropathy, myelitis, encephalopathy, coma, and even death. Usually, the nematodes die in the central nervous system (*6*).

Neuroangiostrongyliasis is a global emerging disease with serious implications for animal and public health (*4*). Globalization has helped disperse, and probably continues to disperse, rat lungworms. Infected rats (and snails) travel by ship, thereby transferring the parasite between continents and countries (*7*). Infected rats have been found near the port of Valencia but also several kilometers from the coast, suggesting a wide distribution of the rat lungworm in the city (Appendix Figure 3).

*A. cantonensis* lungworms have been reported widely in Asia, Africa, and America. However, in Europe, they have thus far been reported exclusively at the insular level, specifically in *R. norvegicus* and *R. rattus* rats in Tenerife (Canary Islands) and in *Atelerix algirus* hedgehogs in Mallorca (Balearic Islands) (*2*,*8*,*9*). Although a possible autochthonous human case of *A. cantonensis* infection was (immunologically) diagnosed in France, the possibility of its being an imported case was not ruled out (*10*).

*A. cantonensis* lungworms, a dangerous invasive species, agents of a potentially fatal emerging infectious disease, are spreading into locations beyond their typical tropical/subtropical distribution, probably favored not only by globalization but also by climate change. Epidemiologic surveys of rat populations in Europe, preferably in urban/peri-urban areas, with the involvement of government entities, pest control agencies, and experts in parasitic zoonoses, should help minimize future potential human infections. 

Appendix Supplemental results from study of autochthonous *Angiostrongylus cantonensis* lungworm in urban rats, Valencia, Spain, 2021.
