# Supplementary material for: Autochthonous Angiostrongylus cantonensis Lungworms in Urban Rats, Valencia, Spain, 2021
Source: Emerg Infect Dis. 2022 Dec;28(12):2564–7. doi: 10.3201/eid2812.220418 (PMC9707565; doi:10.3201/eid2812.220418)
Supplement: Appendix — Supplemental results from study of autochthonous Angiostrongylus cantonensis lungworm in urban rats, Valencia, Spain, 2021. [file 22-0418-Techapp-s1.pdf]

Article DOI: https://doi.org/10.3201/eid2812.220418

Autochthonous *Angiostrongylus cantonensis* Lungworms in Urban Rats, Valencia, Spain, 2021

Appendix

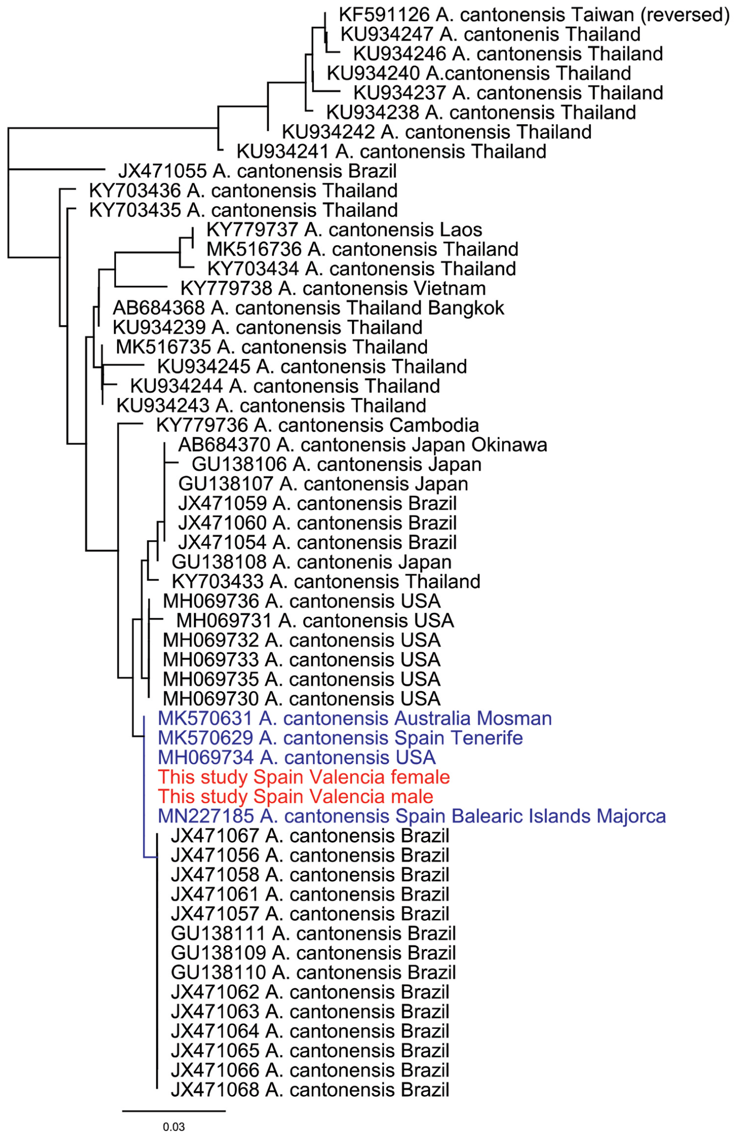

Appendix Figure 1. Neighbor-joining tree based on the sequence of the cytochrome c oxidase subunit 1 (COI) from a female *Angiostrongylus cantonensis* specimen from *Rattus norvegicus* and a male from *Rattus rattus.*

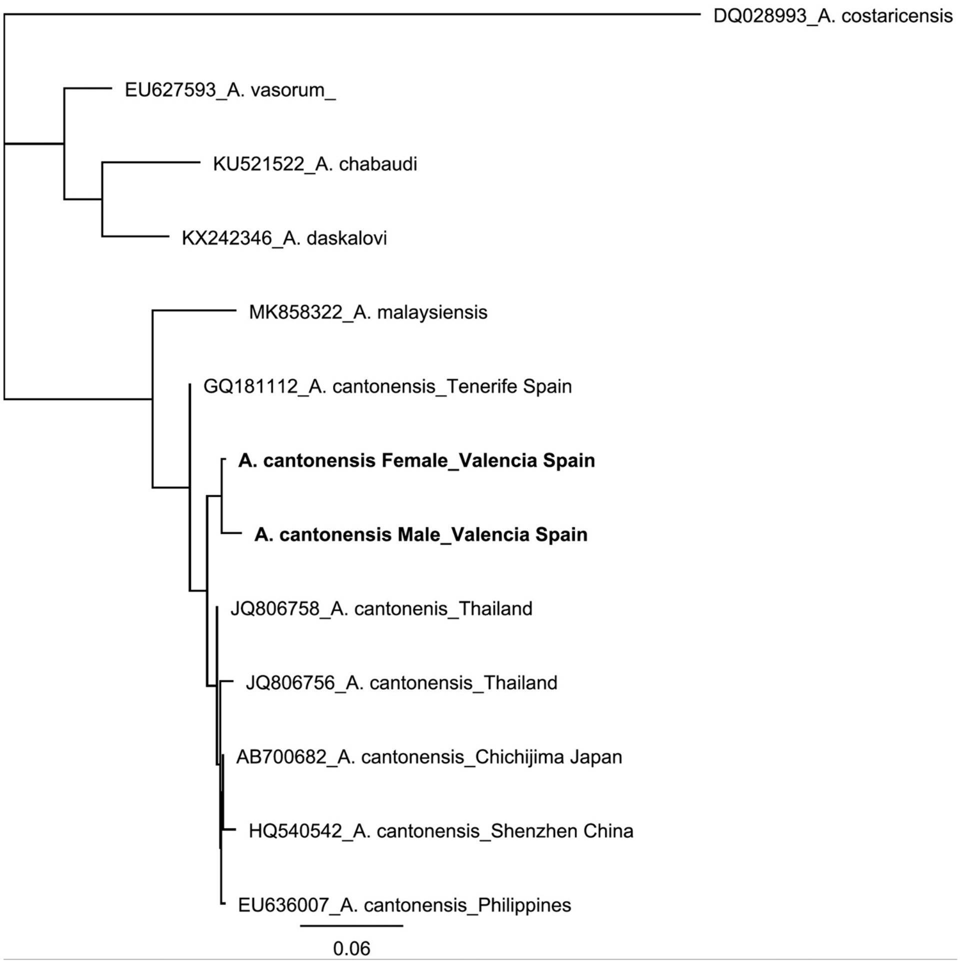

Appendix Figure 2. Neighbor-joining tree based on the sequence from the second internal transcribed spacer (ITS-2) gene region from two nematode specimens from *Rattus norvegicus* and *Rattus rattus,* respectively.

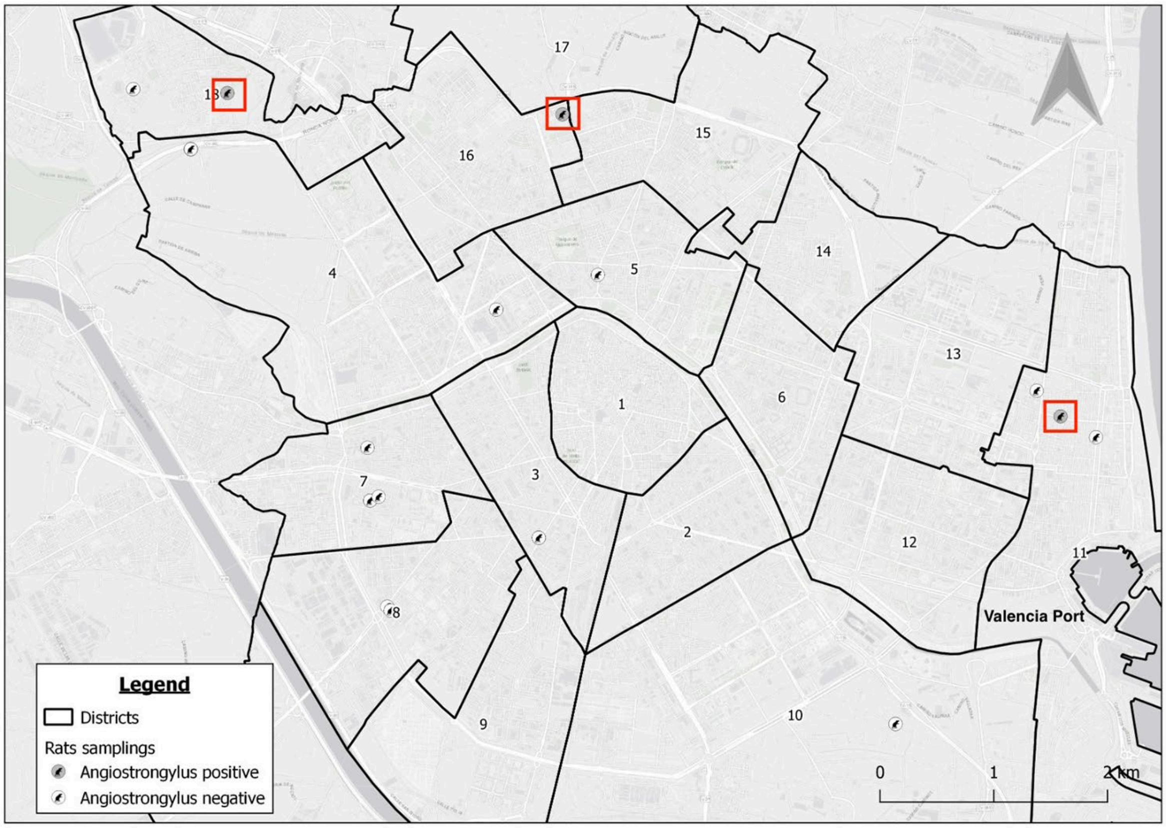

Appendix Figure 3. Map of the districts of Valencia showing the trapping sites of the 27 studied rats. Infected rats were trapped in districts 11, 16 and 18.
